# MiR-30a-5p confers cisplatin resistance by regulating IGF1R expression in melanoma cells

**DOI:** 10.1186/s12885-018-4233-9

**Published:** 2018-04-11

**Authors:** Yuxia Li, Jie Zhang, Yajing Liu, Bingyue Zhang, Fubo Zhong, Shubin Wang, Zhengyu Fang

**Affiliations:** 1 Biomedical Research Institute, Shenzhen Peking University-The Hong Kong University of Science and Technology Medical Center, No. 1120 Lianhua Road, Futian District, Shenzhen, Guangdong province China; 2 Department of Medical Oncology, Peking University Shenzhen Hospital, No. 1120 Lianhua Road, Futian District, Shenzhen, Guangdong Province China

**Keywords:** Melanoma, Cisplatin, miR-30a-5p, IGF1R, Chemo-resistance

## Abstract

**Background:**

Melanoma is notoriously resistant to all current modalities of cancer therapies including chemotherapy. In recent years, microRNAs (miRNAs) have emerged as molecular regulators in the development and progression of melanoma. However, the relationship between microRNA and chemo-resistance of melanoma is little known. In present study, we aimed to investigate the miRNAs related to cisplatin-resistance in melanoma cells.

**Methods:**

After cisplatin (DDP) resistant melanoma cells (M8/DDP and SK-Mel-19/DDP) were established in-vitro, high-throughput screening of differentially expressed miRNAs between resistant cells and parental cells were performed.

**Results:**

It was found that a cancer-related miRNA, miR-30a-5p, was highly over-expressed in resistant cells. Transfection of miR-30a-5p mimic or inhibitor could alter the sensitivity of melanoma cells to cisplatin. Next, we showed that Insulin Like Growth Factor 1 Receptor (IGF1R) gene turned out to be a direct target of miR-30a-5p. Knockdown of IGF1R in melanoma cells could not only reduce the sensitivity to cisplatin but also lead to cell cycle arrest by regulating phosphorylation of Serine-Threonine Protein Kinase (P-AKT (Ser473)) and Tumor Protein P53 (P53).

**Conclusion:**

Taken together, our study demonstrated that miR-30a-5p could influence chemo-resistance by targeting IGF1R gene in melanoma cells, which might provide a potential target for the therapy of chemo-resistant melanoma cells.

## Background

Melanoma is developed from melanocytes, and is the world’s most aggressive skin cancer [1]. Although highly effective new treatment strategies have been gradually used, chemotherapy is still a major treatment for melanoma [2]. Cisplatin is one of the most commonly used chemotherapeutic agents for melanoma. However, the lack of response due to cisplatin resistance has been a main problem that affects the outcome of melanoma therapy. Thus, adjuvant therapy to enhance cisplatin efficiency has become an important chemotherapeutic strategy.

MiRNA is a kind of 20-24 nt non-coding single strand RNA that has been shown to extensively involve in the translational inhibition which is relevant to several physiological and pathological processes. The mechanism of their action is mainly based on the incomplete matching between the seed region of the 5’end of the 21-23 nt sequence and the partial complementary sequence of the 3′ untranslated region(3’-UTR) of target mRNAs [3–5]. Recent studies have suggested that chemo-resistance of cancer cells might be modulated via the changes of miRNA levels [6–11]. For instance, miR-92b is significantly up-regulated in lung cancer cells and knockdown of miR-92b inhibits cell growth and sensitizes A549/DDP cells to DDP by targeting phosphatase and tensin homolog (PTEN) [12]. MiR-26b is prominently down-regulated in DDP-resistant nasopharyngeal carcinoma cells and induces cisplatin resistance by repressing Jagged 1(JAG1) [13]. In Hep-2 laryngeal cancer stem cells, osteosarcoma cells and ovarian cancer cells, miR-125a, miR-497 and miR-199a are able to reverse cisplatin resistance respectively [14–16].

Till now, there is few report about the role of miRNAs in the chemo-resistance of melanoma cells. In present study, we constructed two cisplatin resistant melanoma cell lines and screened miRNAs that related to cisplatin resistance. Here we reported that miR-30a-5p was over-expressed in cisplatin resistant melanoma cells and could influence the activity of PI3K/AKT and the protein level of P53 by targeting IGF1R gene. These results may provide new insights into the molecular mechanisms of cisplatin resistance in melanoma cells and novel therapeutic targets to cure chemo-resistant melanoma.

## Methods

### Cell culture

Human melanoma cancer cells (M8, M8/DDP, SK-Mel-19 and SK-Mel-19/DDP) and HEK293T cells were obtained from Jennio Biotech. (Jennio, China). All cells were cultured in Dulbecco Modified Eagle Medium (DMEM, Gibco, USA) supplemented with 10% fetal bovine serum (FBS, Gibco, USA), 100 IU/ml penicillin (Gibco, USA), and 100 μg/ml streptomycin (Gibco, USA) in a humidified atmosphere containing 5% CO_2_ at 37 °C, and subcultured every 3 days at 10–30% confluence. To establish cisplatin-resistant M8/DDP and SK-Mel-19/DDP cells, M8 and SK-Mel-19 cells were first treated with 0.52 μmol/L (μM) of cisplatin (Hansoh, China), and then were treated with increased concentrations of cisplatin in a stepwise manner during each passage. To maintain the drug-resistance phenotype, cisplatin (with the final concentration of 33.4 μM) was added to the culture media for M8/DDP and SK-Mel-19/DDP cells.

### Drug sensitivity assay

Human melanoma cancer cells M8, M8/DDP, SK-Mel-19 and SK-Mel-19/DDP were plated in 6-well plates (3 × 10^5^ cells/well) and 100 pmol of the miR-30a-5p mimic or mimic control were transfected into M8 and SK-Mel-19 cells, while 100 pmol of the miR-30a-5p inhibitor or inhibitor control were transfected into the M8/DDP and SK-Mel-19/DDP, using Lipofectamine-2000 (Invitrogen, USA) according to the manufacturer’s protocol. 24 h after medium changed, the cells were seeded in 96-well plates (M8 and M8/DDP were 4 × 10^3^ cells/well; SK-Mel-19 and SK-Mel-19/ DDP were 5 × 10^3^ cells/well) for the following experiment. After cell adhesion, cisplatin was added at a final concentration of 4.18, 8.35, 16.7, 33.4, 66.8 μM to cells. 72 h after the addition of the drug, cell viability was assessed by 3-(4,5-dimethylthiazol-2-yl)-5-(3-carboxymethoxyphenyl)-2-(4-sulfophenyl)-2H-tetrazolium, inner salt (MTS, Promega, USA) assay according to the manufacturer’s protocol. The absorbance at 490 nm (A490) of each well was read on iMark Microplate Absorbance Reader (Bio-Rad, USA).

### Real-time quantitative PCR

Total RNA was isolated from cells at the logarithmic phase using Trizol (Sigma, USA) according to the manufacturer’s protocol. First-strand cDNA was synthesised using GoScript™ Reverse Transcription System Kit (Promega, USA). Real-time PCR was performed with GoTaq qPCR Master Mix (Promega, USA) using a C1000 Thermal Cycler apparatus (Bio-Rad) in 20 μl reaction volume according to manufacturer’s protocols. The procedure was as follows: 95 °C-3 min; 39 × (95 °C-15 s, 60 °C-60 s,72 °C-30 s, for mRNA; 95 °C-15 s, 60 °C-60 s for miRNA); 95 °C-10 s, followed by a melt curve analysis (60 °C to 95 °C, increment 0.5 °C for 20 s) to confirm specificity of the PCR primers. Threshold cycle (Ct)-values for mRNA and miRNA species were normalized to housekeeping genes: GADPH for mRNAs and U6 for mature miRNAs (Primers were listed in Table 1). The fold change was calculated using the 2^-ΔΔCt^ Method.

**Table 1:** Primers list

Primer name	Sequence (5′ to 3′)
has-miR-10b-5p-F	GCCATTACCCTGTAGAACCG
has-miR-10b-5p-RT	GTCGTATCCAGTGCGTGTCGTGGAGTCGGCAATTGCACTGGATACGACCACAAA
has-miR-1246-F	CGGGAGAATGGATTTTTGG
has-miR-1246-RT	GTCGTATCCAGTGCGTGTCGTGGAGTCGGCAATTGCACTGGATACGACCCTGCT
has-miR-138-5p-F	GCGAAGCTGGTGTTGTGAA
has-miR-138-5p-RT	GTCGTATCCAGTGCGTGTCGTGGAGTCGGCAATTGCACTGGATACGACCGGCCT
has-miR-146a-5p-F	CGGCGGCTGAGAACTGAAT
has-miR-146a-5p-RT	GTCGTATCCAGTGCAGGGTCCGAGGTATTCGCACTGGATACGACAACCCA
has-miR152-3p-F	CGGCTCAGTGCATGACAGA
has-miR152-3p-RT	GTCGTATCCAGTGCGTGTCGTGGAGTCGGCAATTGCACTGGATACGACCCAAGT
has-miR-15a-3p-F	CCGTCCAGGCCATATTGTG
has-miR-15a-3p-RT	GTCGTATCCAGTGCAGGGTCCGAGGTATTCGCACTGGATACGACTGAGGC
has-miR-181a-3p-F	CAGCACCATCGACCGTTGA
has-miR-181a-3p-RT	GTCGTATCCAGTGCGTGTCGTGGAGTCGGCAATTGCACTGGATACGACGGTACA
has-miR-181a-5p-F	GACGCAACATTCAACGCTG
has-miR-181a-5p-RT	GTCGTATCCAGTGCAGGGTCCGAGGTATTCGCACTGGATACGACACTCAC
has-miR-182-5p-F	CGGATTTGGCAATGGTAGA
has-miR-182-5p-RT	GTCGTATCCAGTGCGTGTCGTGGAGTCGGCAATTGCACTGGATACGACAGTGTG
has-miR-193a-5p-F	ATCTATGGGTCTTTGCGGG
has-miR-193a-5p-RT	GTCGTATCCAGTGCGTGTCGTGGAGTCGGCAATTGCACTGGATACGACTCATCT
has-miR-210-3p-F	CAGTGGCTGTGCGTGTGAC
has-miR-210-3p-RT	GTCGTATCCAGTGCGTGTCGTGGAGTCGGCAATTGCACTGGATACGACTCAGCC
has-miR-21-3p-F	ACGGCACCAACACCAGTCG
has-miR-21-3p-RT	GTCGTATCCAGTGCAGGGTCCGAGGTATTCGCACTGGATACGACACAGCC
has-miR-301a-3p-F	AGGCGGCAGTGCAATAGTA
has-miR-301a-3p-RT	GTCGTATCCAGTGCGTGTCGTGGAGTCGGCAATTGCACTGGATACGACGCTTTG
has-miR-30a-5p-F	TGCCGTGTAAACATCCTCG
has-miR-30a-5p-RT	GTCGTATCCAGTGCGTGTCGTGGAGTCGGCAATTGCACTGGATACGACCTTCCA
has-miR-449b-3p-F	GCGTCAGCCACAACTACCC
has-miR-449b-3p-RT	GTCGTATCCAGTGCGTGTCGTGGAGTCGGCAATTGCACTGGATACGACAGTGGC
has-miR-454-5p-F	CAGCGGCACCCTATCAATA
has-miR-454-5p-RT	GTCGTATCCAGTGCAGGGTCCGAGGTATTCGCACTGGATACGACGCAGAG
has-miR-4701-3p-F	CCAGATGGGTGATGGGTGT
has-miR-4701-3p-RT	GTCGTATCCAGTGCAGGGTCCGAGGTATTCGCACTGGATACGACACACCA
URP-1	GTGTCGTGGAGTCGGCAA
URP-2	CGCAGGGTCCGAGGTATTC
U6-F	CTCGCTTCGGCAGCACA
U6-R	AACGCTTCACGAATTTGCGT
IGF1R-F	GGCTGGGGCTCTTGTTTACC
IGF1R-R	GCCTCTCTCGAGTTCGCC
GAPDH-F	TCCAAAATCAAGTGGGGCGA
GAPDH-R	TGATGACCCTTTTGGCTCCC
TuD-miR-30a-5p-R	CGCGAAAAAAGACGGCGCTAGGATCATCTTGTGTAAACATCAGATCTCGACTGGAAGGTTGTATTCTGTGACCAGAATACTTGTGTAAACATCAGATCTCGACTGGAAGGTTGATGATCCTAGCGCCGTCG
IGF1R-wt-F	AATTCTAGCGATCGCTCGAG TCGCACTCGTCAGTTGTCAGTT
IGF1R-wt-R	ATTTTATTGCGGCCAGCGGCCGCATCCTTTTTTGGCATATTGTAAAGC
IGF1R-mut A-F	CGAAGATCTGCAAATGTAGAACTAATTAAATGTTTCATTGCATTT
IGF1R-mut A-R	ACATTTGCAGATCTTCGTCAACTGACTACCCG
IGF1R-mut B-F	GACACCACAAATGTAGCTAGCTTTACAATATGCCAAAAA
IGF1R-mut B-R	GCTACATTTGTGGTGTCCTAAAAAAAAAAAAAAAAAA

### Western blot analysis

Cells were lysed in SDS lysis buffer (Beyotime, China) supplemented with protease inhibitor cocktail (Takara, China). Total protein was quantified by BCA Protein Assay Kit (Beyotime, China), and proteins (10 μg/per lane) were separated by SDS–PAGE and transferred onto a polyvinylidene fluoride (PVDF) membrane (Pall, USA). The membranes were blocked in bovine serum albumin (BSA) (3% *w*/*v* in PBS + 0.1% w/v in Tween 20) for 30 min at room temperature and incubated with diluted antibodies at 4 °C overnight. Proteins were detected by enhanced chemiluminescence system (Pierce, USA) according to the manufacturer’s instructions. Data were normalized to GAPDH.

### Plasmid construction and lentiviral infection

To knock down miR-30a-5p expression, TuD-miR-30a-5p was constructed based on the Tough Decoy (TuD) design [17]. Oligonucleotides of the Tough Decoy RNA were annealed and cloned into BamHI and MluI site of lentiviral vector pLent-U6-GFP-puro (ViGene, China), resulting in TuD-miR-30a-5p being driven by polymerase III promoter U6. Lentivirus was produced by transfecting HEK293T cells with each lentiviral construct together with the packaging vectors psPAX2 and pMD2.G (ViGene, China) using Lipofectamine-2000 (Introvigen, USA) according to the instructions of the manufacturer. The supernatant was collected 72 h after transfection and was centrifuged (4000 g for 5 min at room temperature) to remove cell debris; the supernatant was used for M8/DDP and SK-Mel-19/DDP cells infection. The infected cells were then selected by supplementing the culture medium with 6 μg/ml of puromycin 48 h after infection. The efficiency of the inhibition of miRNAs was confirmed by real-time PCR analysis.

### Dual luciferase reporter assays

Based on the miRNA databases (microRNA.org, miRDB and TargetScan databases), IGF1R is a predicted target of miR-30a-5p. Hence, we cloned IGF1R 3’-UTR fragment containing the predicted site (5’-GTTTACA-3′ and 5’-TGTTTAC-3′) or the mutant sequence (5’-CAAATGT-3′ and 5’-ACAAATG-3′) into psiCHECK™-2 luciferase reporter vector (Promega, USA) (Primers were listed in Table 1). For luciferase assay, the reporter plasmid was co-transfected with miR-30a-5p mimic or mimic control in HEK293T cells. After 48 h, cells were lysed and luciferase expression was measured using the Dual-luciferase assay system (Promega, USA) following the manufacturer’s protocol. The renilla luciferase (Rluc) was normalized by the firefly luciferase (Luc).

### Data analysis

GraphPad Prism software (La Jolla, CA) was used to plot the curves and statistical analysis. Data were presented as mean ± SD from at least three independent experiments. Statistical analysis was performed by Student’s *t* test. *p* values of < 0.05 (*), < 0.01(**), and < 0.001 (***) were considered significant.

## Results

### MiR-30a-5p is highly expressed in cisplatin-resistant melanoma cells

**Fig. 1:**
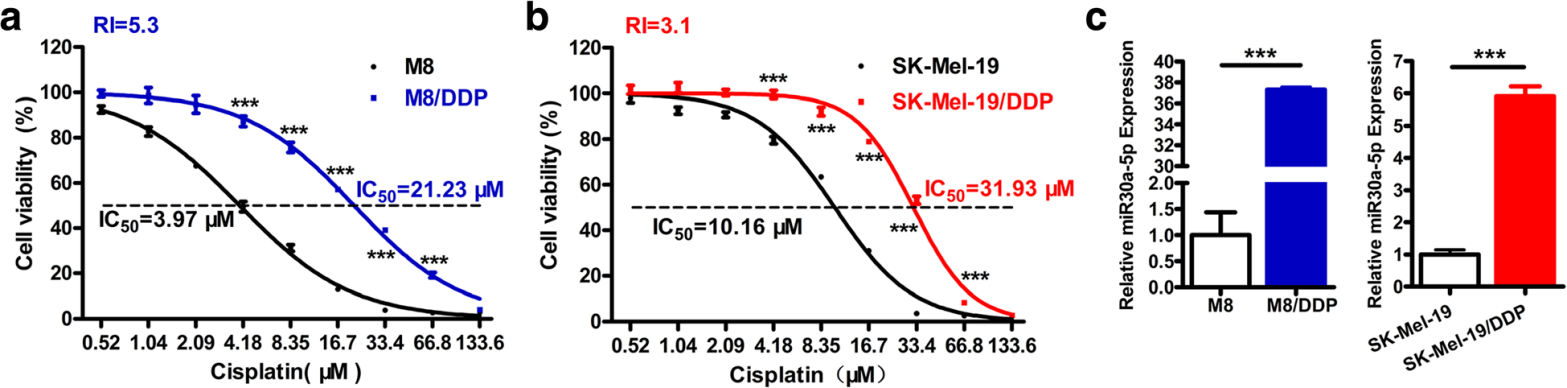
MiR-30a-5p is highly expressed in cisplatin-resistant melanoma cells. **a**, **b** M8, M8/DDP, SK-Mel-19 and SK-Mel-19/DDP cells were treated with indicated concentrations of cisplatin for 72 h and then were subjected to MTS assay. **c** The mRNA expression level of miR-30a-5p in M8, M8/DDP, SK-Mel-19 and SK-Mel-19/DDP cells was detected by real-time PCR analysis

Two cisplatin-resistant cell lines M8/DDP and SK-Mel-19/DDP were induced by continuous exposure to cisplatin after 5 months for more than 50 cell passages. The cell lines were used for experiments after culturing in drug-free medium for another 2 months. We then tested the half maximal inhibitory concentration (IC_50_) and drug resistance indices (RI) of the resistant cells as well as their parental cells by MTS assay. In Fig. 1a and b, the IC_50_ of M8 cells was 3.97 μM, the IC_50_ of M8/DDP cells was 21.23 μM, the resistance index was 5.3; the IC_50_ of SK-Mel-19 cells was 10.16 μM, the IC_50_ of SK-Mel-19/DDP cells was 31.93 μM, and its resistance index was 3.1. The results indicated that the resistant lines were established successfully. Since the drug-resistant cells differed significantly from their parental cells at cisplatin concentrations of 4.18 μM, 8.35 μM, 16.7 μM, 33.4 μM, and 66.8 μM, these five concentrations were selected for follow-up experiments.

We used microRNA microarray analysis to screen the differential expressed miRNAs (≥2.0 fold or ≤ 0.5 fold) between the resistant cells and their parental cells, and 21 miRNAs were verified by real-time PCR and listed in Table 2. Among them, a cancer-related miRNA, miR-30a-5p, was found to be markedly up-regulated in both M8/DDP and SK-Mel-19/DDP cells. This difference in expression of miR-30a-5p was also validated by real-time PCR analysis (Fig. 1c), the difference multiples of miR-30a-5p were 35.37 and 5.65.

**Table 2:** Differential Expressed miRNAs between resistant cells and the parental cells

miRBase accession No.	miRNA	M8/DDP vs M8		SK-Mel-19/DDP vs SK-Mel-19	
		Change	*P*-value	Change	*P*-value
MIMAT0000254	hsa-miR-10b-5p	0.62	0.04	0.86	0.09
MIMAT0005898	hsa-miR-1246	0.35	0.04	1.42	0.25
MIMAT0000430	hsa-miR-138-5p	1.33	0.18	3.00	0.83
MIMAT0000449	hsa-miR-146a-5p	0.78	0.04	1.70	0.04
MIMAT0000438	hsa-miR-152-3p	0.39	0.02	3.45	0.50
MIMAT0004488	hsa-miR-15a-3p	1.17	0.13	0.59	0.14
MIMAT0000270	hsa-miR-181a-3p	1.57	0.03	1.70	0.38
MIMAT0000256	hsa-miR-181a-5p	2.19	0.06	2.50	0.49
MIMAT0000259	hsa-miR-182-5p	0.94	0.03	1.30	0.07
MIMAT0004614	hsa-miR-193a-5p	0.77	0.07	0.53	0.05
MIMAT0000267	hsa-miR-210-3p	1.77	0.15	0.61	0.08
MIMAT0004494	hsa-miR-21-3p	0.35	0.06	0.64	0.20
MIMAT0000688	hsa-miR-301a-3p	1.35	0.29	1.12	0.34
MIMAT0000087	hsa-miR-30a-5p	35.37	0.06	5.65	0.85
MIMAT0009203	hsa-miR-449b-3p	2.43	0.68	0.95	0.29
MIMAT0003884	hsa-miR-454-5p	3.12	0.10	0.79	0.16
MIMAT0019799	hsa-miR-4701-3p	1.20	0.35	1.33	0.09
MIMAT0003304	hsa-miR-634	2.83	0.23	0.80	0.01
MIMAT0027433	hsa-miR-6766-3p	1.07	0.08	1.09	0.32
MIMAT0027459	hsa-miR-6779-3p	1.75	0.65	1.09	0.44
MIMAT0027484	hsa-miR-6792-5p	1.59	0.46	0.76	0.14

### MiR-30a-5p affects the resistance of melanoma cells to cisplatin

**Fig. 2:**
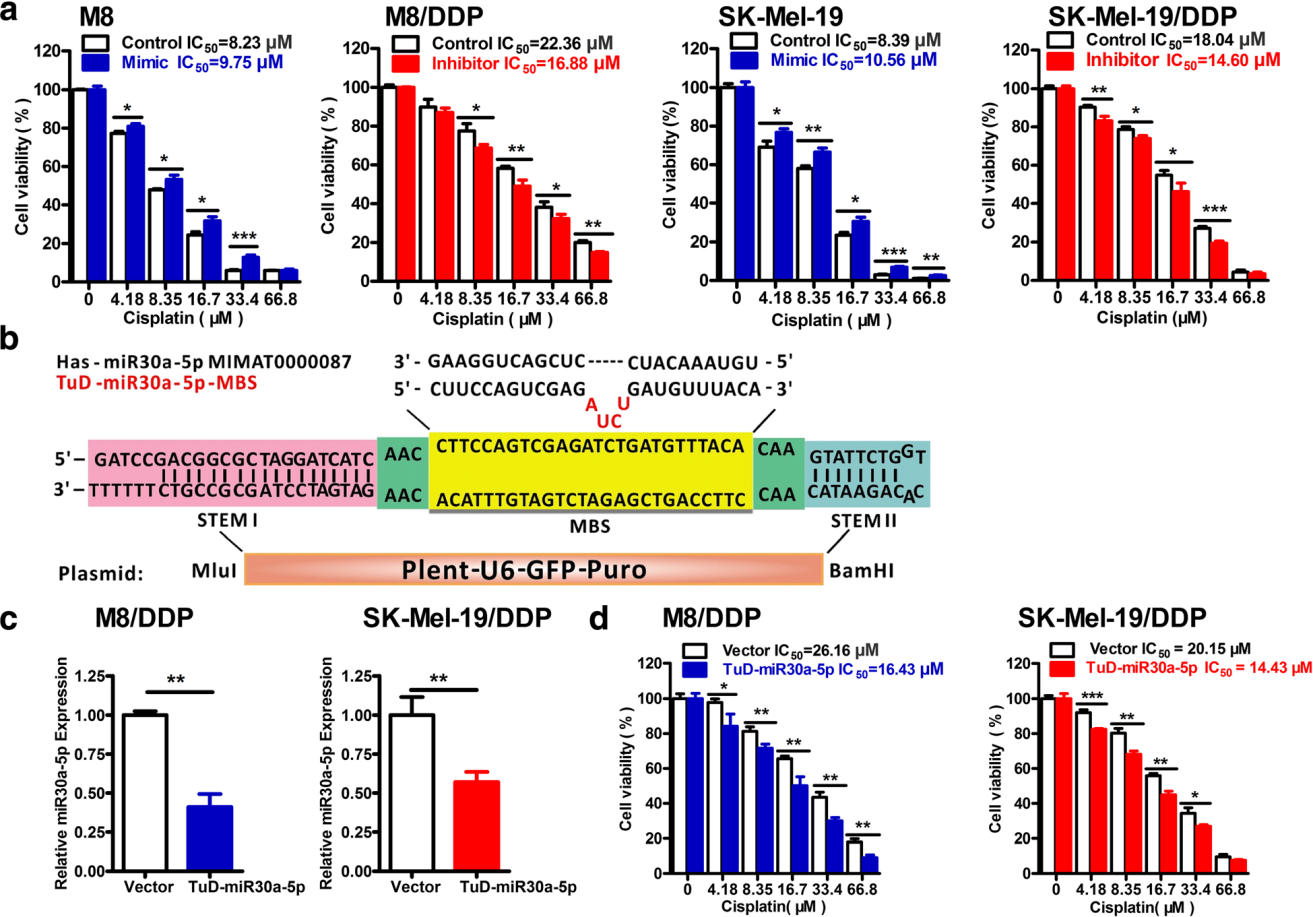
MiR-30a-5p affects the resistance of melanoma cells to cisplatin. **a** After 12 h of transfection miR-30a-5p mimic or inhibitor, cells were incubated with the indicated concentrations of cisplatin for 72 h and subsequently subjected to MTS assay. **b** Construction of the Plent-U6-GFP-Puro vector expressed TuD-miR-30a-5p. **c** The knockdown efficiency of miR-30a-5p in M8/DDP and SK-Mel-19/DDP cells was detected by real-time PCR. **d** MiR-30a-5p knockdown and vector control cells were treated with different concentrations of cisplatin for 72 h. MTS assay was used to detect cell proliferation

To explore the potential roles of miR-30a-5p in the cisplatin-resistance of melanoma cells, in-vitro studies using miR-30a-5p mimics and inhibitors were performed. As shown in Fig. 2a, the IC_50_ of M8 cells increased when transfected with miR-30a-5p mimic, which indicated their resistance to cisplatin was enhanced. Similar results were obtained in SK-Mel-19 cells. On the other hand, the IC_50_ of M8/DDP cells was markedly reduced after transfected with miR-30a-5p inhibitor, which could also be repeated in the SK-Mel-19/DDP cells.

Transfection into a cell can be transient or stable. To further study the relationship between miR-30a-5p and cisplatin resistance in melanoma, we constructed a Tough Decoy (TuD) vector expressing a miR-30a-5p-TuD that could stably suppress the expression of miR-30a-5p in M8/DDP and SK-Mel-19/DDP cells (Fig. 2b). Knockdown efficiency of the stable transfection was validated by the real-time PCR analysis (Fig. 2c), the transfection of TuD-miR-30a-5p reduced the expression of miR-30a-5p by more than 40% in M8/DDP and SK-Mel-19/DDP cells. As shown in Fig. 2d, the IC_50_ of M8/DDP and SK-Mel-19/DDP cells decreased significantly after stably transfected with TuD-miR-30a-5p. In both transient transfection and stable transfection approaches, the sensitivity of the melanoma cells to cisplatin was influenced by the change of miR-30a-5p expression. These provide the evidence of the association between miR-30a-5p and cisplatin-resistance of the melanoma cells.

### The insulin like growth factor 1 receptor (IGF1R) is a direct target of miR-30a-5p

**Fig. 3:**
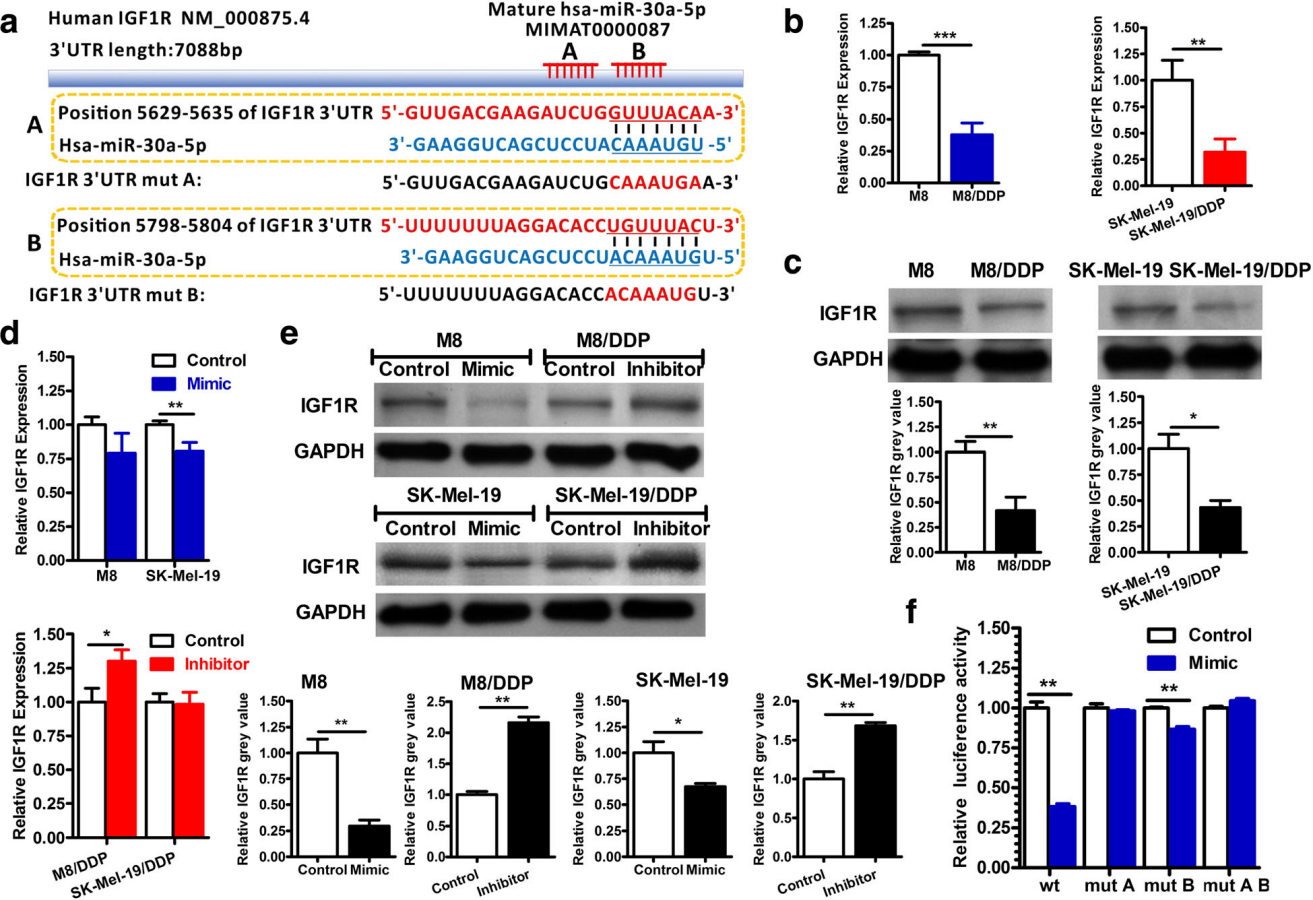
IGF1R is a direct target of miR-30a-5p. **a** The 3’-UTR of IGF1R contains two predicted miR-30a-5p binding sites. The alignment of the seed regions of miR-30a-5p with IGF1R 3’-UTRs is shown in the figure. The sites of target mutagenesis are indicated in red underline. **b** The mRNA expression level of IGF1R in M8, M8/DDP, SK-Mel-19 and SK-Mel-19/DDP cells was detected by real-time PCR analysis. **c** The protein expression level of IGF1R in M8, M8/DDP, SK-Mel-19 and SK-Mel-19/DDP cells was detected by Western blotting analysis. **d** After 24 h of transfection miR-30a-5p mimic or inhibitor in M8, M8/DDP, SK-Mel-19 and SK-Mel-19/DDP cells, the expression of IGF1R was detected by real-time PCR. **e** After 48 h of transfection miR-30a-5p mimic or inhibitor in M8, M8/DDP, SK-Mel-19 and SK-Mel-19/DDP cells, the expression of IGF1R was detected by Western blotting analysis. **f** HEK293T cells were co-transfected with psiCHECK-2 reporter containing a wild type or mutated miR-30a-5p binding site shown in Fig. 3a together with control or miR-30a-5p mimic. Luciferase activities were measured after 24 h of transfection

Identification of miRNA-regulated gene targets is a necessary step to understand miRNA functions. First, we used the online tools (microRNA.org, miRDB and TargetScan databases) to screen the potential target genes of miR-30a-5p. Among the genes obtained from screening results, we noticed that a cancer-related gene, IGF1R, might be a tentative target of miR-30a-5p whose 3’-UTR contains two putative miR-30a-5p seed sites (Fig. 3a). We noticed that the mRNA and protein levels of IGF1R in M8/DDP and SK-Mel-19/DDP were significantly lower than those in the parental cells (Fig. 3b & c), which were contrary to the expression levels of miR-30a-5p in the two groups. In the in-vitro approaches, transfection of miR-30a-5p mimics could reduce the protein levels of IGF1R in M8 and SK-Mel-19 cells, while transfection of miR-30a-5p inhibitors could promote the expression of IGF1R in the resistant cells. At the same time, we noticed that the mRNA levels of IGF1R were less influenced by the alteration of miR-30a-5p expression (Fig. 3d and e), which suggested that miR-30a-5p could not induce the degradation of IGF1R mRNA.

To test whether the predicted miR-30a-5p target sites in the 3’-UTR of IGF1R mRNA were responsible for its regulation, we cloned IGF1R 3’-UTR wild type (wt) or 3’-UTR mutant types (mut A, mut A and mut A B) into downstream of the luciferase reporter gene. In the absence of miR-30a-5p, luciferase would be expressed, whereas in the presence of miR-30a-5p, luciferase mRNA would be degraded. As shown in Fig. 3f, when the double luciferase reporter vector containing the wild type of IGF1R 3’-UTR and miR-30a-5p mimic were co-transfected into HEK293T cells, the Renilla luciferase activities were significantly inhibited. Mutations of the IGF1R 3’-UTR binding site in region A drastically abolished ability of miR-30a-5p to regulate luciferase expression, while mutations in the region B partially impaired the luciferase expression. Thus, miR-30a-5p could directly bind to both region A and B on 3’-UTR of IGF1R gene.

### IGF1R can influence cell cycle of melanoma cells by regulating AKT activity and protein level of P53

**Fig. 4:**
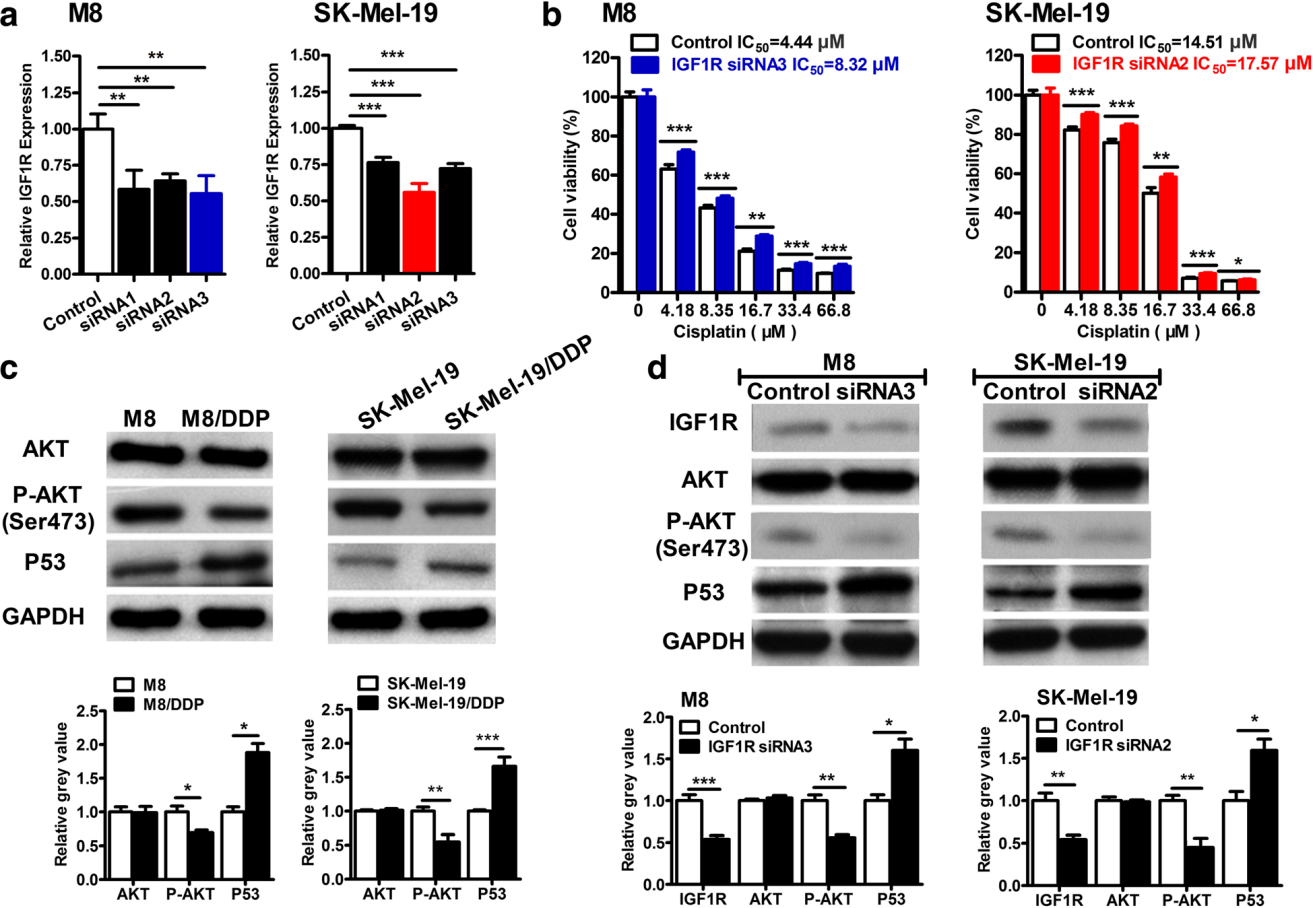
IGF1R can regulate AKT activity and protein level of P53. **a** IGF1R siRNAs were transfected into M8 and SK-Mel-19 cells for 24 h, then the mRNA expression level of IGF1R was detected by real-time PCR. **b** After 12 h of silencing IGF1R, cells were incubated with the indicated concentrations of cisplatin for 72 h and subsequently subjected to MTS assay. **c** The protein level of AKT, P-AKT, P53 in M8, M8/DDP, SK-Mel-19 and SK-Mel-19/DDP cells. **d** IGF1R siRNAs were transfected into M8 and SK-Mel-19 cells for 48 h, then the protein levels of IGF1R, AKT, P-AKT, P53 were detected by Western blotting analysis

We next investigated whether IGF1R itself could influence cisplatin resistance in the two melanoma cell lines. We conducted small interfering RNA (siRNA)-based silencing of IGF1R and assessed its efficiency in M8 and SK-Mel-19 cells. As shown in Fig. 4a, siRNA3 was more efficient in M8 cells and siRNA2 was more efficient in SK-Mel-19 cells. After transfection these two small interfering RNA, the expression of miR-30a-5p reduced more than 40% in M8 and SK-Mel-19 cells. Then, after IGF1R was knockdown in the M8 and SK-Mel-19 cells using the appropriate siRNAs, the sensitivities of both cell lines to cisplatin were reduced (Fig. 4b).This suggested the direct correlationship between IGF1R and cisplatin-resistance in melanoma cells.

**Fig. 5:**
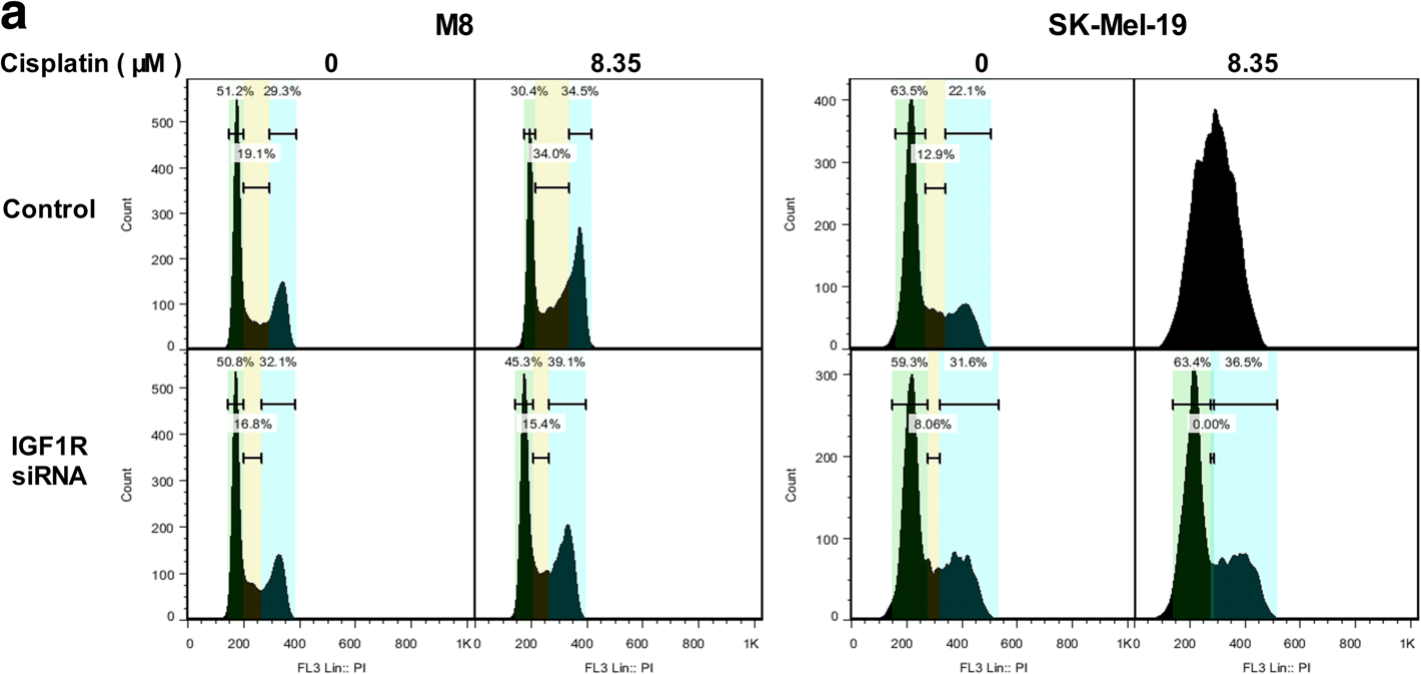
IGF1R can influence cell cycle of melanoma cells. **a** After silencing IGF1R, cells were treated with cisplatin at 0, 8.35 μM for 24 h, and the cell cycle of M8 and SK-Mel-19 cells was detected by flow cytometry

As a receptor of insulin-like growth factor, IGF1R has tyrosine kinase activity and plays a critical role in transformation events. Previous studies have shown that activation of the IGF1R signaling pathway is involved in proliferation, survival, and metastasis of cancer cells. In the present study, we noticed that the expression of phosphorylation of AKT (Ser473) was lower while the expression of P53 protein was higher in the resistant cells compared to their parental cells (Fig. 4c). We wondered whether it was associated with the alteration of IGF1R. In the subsequent in-vitro studies, we used the specific siRNA to knockdown the expression of IGF1R in M8 and SK-Mel-19 cells, and found that the phosphorylation of AKT (Ser473) was impaired while the protein levels of P53 were increased (Fig. 4d). At the same time, cell cycle arrests were detected in both cell lines (Fig. 5a). Based on the IC_50_ of M8 and SK-Mel-19 cells (Fig. 1a and b, Fig. 4b), we selected three concentrations of cisplatin(4.18 μM, 8.35 μM and 16.7 μM) that cover the range of IC_50_ of the both cell lines for cell cycle examination. Interestingly, we found that cisplatin at concentration of 8.35 μM influence the cell cycle most significantly in the both cell lines. As shown in Fig. 5a, after M8 cells were treated with cisplatin, a G2/M arrest was induced. When the IGF1R was silenced before the cisplatin treatment, the G2/M arrest was impaired. On the other hand, in case of SK-Mel-19 cells, cisplatin treatment induced cell cycle disruption. However, when the IGF1R was silenced before the cisplatin treatment, the cell cycle only varied a little after cisplatin treatment. Thus, IGF1R might play a protective role in cisplatin-mediated DNA damage through regulating cell cycle by influencing AKT/P53 pathway in melanoma cells.

## Discussion

Here we identified a novel miR-30a-5p/IGF1R/AKT/P53 signaling axis that regulates melanoma cells in cisplatin resistance. Our data provide a first insight into the function of miR-30a-5p in regulating cisplatin resistance. Because resistance to cisplatin treatment is one of major causes for chemotherapy failure in treating melanoma [18–20], the discovery of miR-30a-5p as a contributing factor of cisplatin resistance and the identification of the regulatory miR-30a-5p/IGF1R/AKT/P53 signaling axis would help design strategies to increase the efficacy of chemotherapeutic treatment of melanoma.

Some studies have shown that miR-30s have been found involved in the regulation of cisplatin-sensitivity of some cancers, where individual members of the miR-30 family assist cancer cells in both cisplatin sensitivity and cisplatin insensitivity [21–23].In the current study, miR-30a-5p is highly expressed in M8/DDP and SK-Mel-19/DDP cells. Transfection of miR-30a-5p mimics in M8 and SK-Mel-19 cells could enhance the cisplatin-resistance of the cells, while knockdown of miR-30a-5p could impair the resistance of M8/DDP and SK-Mel-19/DDP cells to cisplatin. These findings suggested that miR-30a-5p did correlate with cisplatin resistance in melanoma.

There are contradictory reports on the relationship of IGF1R and acquired resistance in many human cancers. In NSCLC (Non-Small Cell Lung Cancer) cell lines, IGF1R activation could contribute to gefitinib resistance and give rise to the failure of the combination therapy [24, 25]. HCC (Hepatocellular Carcinoma) cells exhibited strong gefitinib resistance and the levels of phosphorylation in IGF1R and AKT were dramatically increased after drug treatment [26]. In advanced hepatocellular carcinoma, activation of IGF1R by ectopic down-regulation of miR-122 counteracted the effects of sorafenib-induced apoptosis, thus conferring sorafenib resistance [27]. MiR-143, miR-503 and miR-1271 modulated cisplatin resistance of human gastric cancer cells by targeting IGF1R [28–30]. These different results could be attributed to different cell contexts, experimental conditions, or assay approaches used in the various studies. In present study, as the target gene of miR-30a-5p, IGF1R is negatively correlated with cisplatin resistance.

IGF1R is a transmembrane receptor tyrosine kinase and can activate multiple downstream signaling cascades, among which the most prominent is the phosphatidylinositol 3 kinase/protein kinase B (PI3K/AKT) pathway [31–33]. PI3-kinase/AKT signaling can act on Mdm2 and thereby negatively regulate P53 [34]. In the present study, transfection of IGF1R-siRNA in M8 and SK-Mel-19 cells not only reduced the sensitivity to cisplatin but also altered the expression of phosphorylated AKT and P53, which subsequently induced cell cycle arrest in both cells. G2/M phase cell arrest here might serve as a protective mechanism following cisplatin-DNA damage, avoiding replication of damaged DNA. And further investigation in other melanoma cell lines is necessary to explore the function and mechanisms of miR-30a-5p in cisplatin resistance of melanoma cells.

In the present study, we obtained these findings only in the cell models. The manifestation of resistance to cisplatin administration has not been related with molecular epidemiological data yet. Next we should concern the actual burden of this resistance mechanism to the human patient population. If there will be clinical trials, we may examine the expression of miR-30a-5p or IGF1R and form of P53 (mutant or wild type) in the patients with cisplatin resistance, and design potential targeted therapeutic approaches.

## Conclusion

To summarize, our studies demonstrate that miR-30a-5p could influence cisplatin-resistance by targeting IGF1R gene and AKT/P53 pathway in melanoma cells, which might provide a potential target for the therapy of chemo-resistant melanoma cells.

## Abbreviations

3’-UTR: 3′ untranslated region
AKT: Serine-Threonine Protein Kinase
BSA: Bovine serum albumin
Ct: Threshold cycle
DDP: Cisplatin
DMEM: Dulbecco modified eagle medium
FBS: Fetal bovine serum
IC_50_: The half maximal inhibitory concentration
IGF1R: Insulin Like Growth Factor 1 Receptor
JAG1: Jagged 1
Luc: Firefly luciferase
miRNA: microRNA
MTS: 3-(4,5-dimethylthiazol-2-yl)-5-(3-carboxymethoxyphenyl)-2-(4-sulfophenyl)-2H-tetrazolium
P53: Tumor Protein P53
PTEN: Phosphatase and tensin homolog
PVDF: Polyvinylidene fluoride
RI: Drug resistance indices
Rluc: Renilla luciferase
TuD: Tough Decoy
μM: μmol/L
